# Hematocolpos: An Uncommon Cause of Acute Urinary Retention and Hydronephrosis

**DOI:** 10.7759/cureus.62737

**Published:** 2024-06-19

**Authors:** Ana I Foles, Rita Carvalho, Inês Oliveira, Sara P Carmo, Jorge Palácios

**Affiliations:** 1 Pediatrics, Unidade Local de Saúde da Arrábida, Setúbal, PRT; 2 Pediatric Surgery, Unidade Local de Saúde da Arrábida, Setúbal, PRT

**Keywords:** hydronephrosis, urinary retention, imperforate hymen, uterovaginal deformity, hematocolpos

## Abstract

Acute urinary retention is uncommon in pediatric age and is usually associated with infection or obstruction of the urinary tract. We present the case of a 12-year-old girl admitted to the emergency room with acute urinary retention and lower urinary tract symptoms. Physical examination revealed abdominal distension and a blue-bulging mass occupying the vaginal introitus. Ultrasound confirmed the diagnosis of hematocolpos and revealed hydronephrosis, caused by compression. Kidney function and urinalysis were normal. A hymen incision and excision of redundant tissue were performed without complications.

Hematocolpos is a condition caused by obstructive uterovaginal deformity. Imperforate hymen is responsible for most of the cases and usually manifests as primary amenorrhea and cyclic lower abdominal pain. The diagnosis of hematocolpos can be challenging. However, it is important to consider it in female adolescents without menarche presenting with acute urinary retention.

## Introduction

Acute urinary retention (AUR) is common in adult urology but is an infrequent condition in pediatric patients, particularly among female patients. A European study estimated an incidence rate of 30.2 per 100,000 pediatric emergency department visits, with only 33% of cases occurring in girls [1]. Acute urinary retention presents as a sudden inability to pass urine, typically associated with lower abdominal pain [2]. Although rare, the potential etiologies behind this condition in female adolescents are diverse. It can be related to infections and inflammatory disorders, such as urinary tract infections or acute vulvovaginitis. Mechanical obstruction, including fecal impaction, urolithiasis, pelvic organ prolapse, hematocolpos, and post-pelvic surgery complications, are also potential causes. Additionally, detrusor underactivity may result from neuropathy, central nervous system disorders, bladder muscle dysfunction, or pharmacological causes. Trauma can also be a contributing factor [1,3].

The management usually includes prompt bladder decompression and analgesia, and subsequent treatment should be directed to the underlying etiology [4]. The following case report represents a rare cause of AUR in pediatric age. It emphasizes the importance of a thorough medical history and physical examination to establish the correct diagnosis.

## Case presentation

We present the case of a 12-year-old female patient admitted to the pediatric emergency department with an eight-hour episode of AUR accompanied by abdominal suprapubic pain, dysuria, and frequent voiding over the past four days. She reported no further symptoms such as fever, nausea, vomiting, flank pain, or recurrent episodes of abdominal pain. The patient was previously healthy, except for a record of constipation. There were no reports of recurrent urinary tract infections, prior surgeries, or recent trauma. Menarche had not yet occurred, and the patient’s family history was unremarkable. Physical examination revealed lower abdominal tenderness and a palpable bladder without any other evident mass. Murphy’s kidney sign was absent. A standard neurologic examination showed no abnormality. Puberty development corresponded to Tanner stage III. At that time, no alteration was noted in the external genitalia examination. Immediate management included bladder decompression via catheterization. Urinalysis was negative for leukocytes, leukocyte esterase, and nitrites, and urine culture was sterile. An abdominal radiography was performed, identifying extensive fecal retention. Fecal disimpaction was accomplished with an effective response. Afterward, the adolescent had two spontaneous urination with symptom resolution. The subsequent abdominal examination was normal, and the patient was discharged. 

Two days later, she returned to the emergency department due to a recurrence of urinary retention, presenting with discomfort in the vulvar region. Physical examination revealed significant abdominal distension, diffuse tenderness, and palpable bladder. Inspection of the external genitalia revealed a blue bulging mass of elastic consistency occupying the entire vaginal introitus. The hypothesis of imperforate hymen causing hematocolpos was considered. Cooperation from the gynecology department was requested, and a urethral catheter was placed.

**Figure 1:**
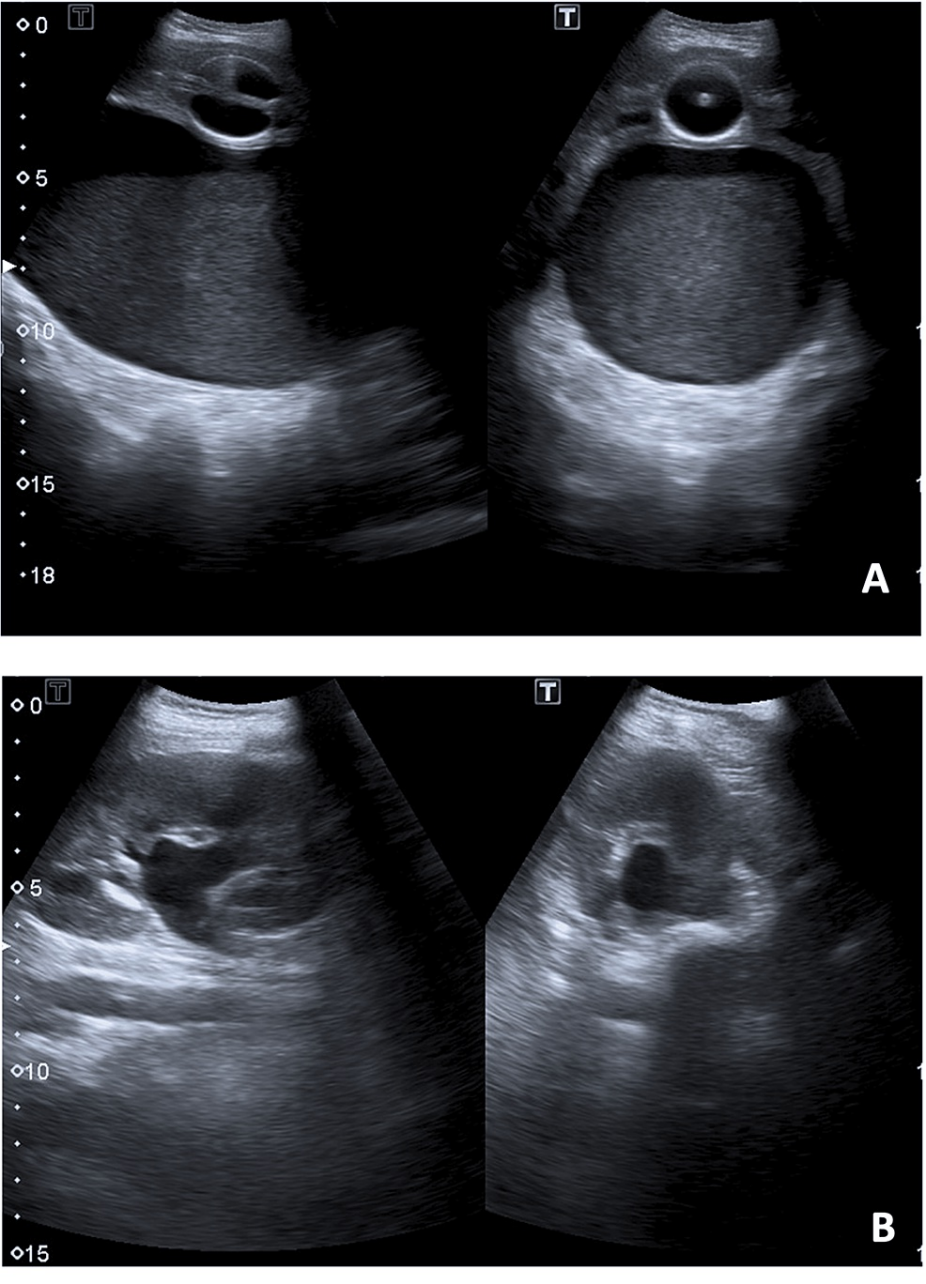
Abdominal and pelvic ultrasound A: Vaginal distension with hemorrhagic content (note: examination performed after bladder catheterization); B: Hydronephrosis

Abdominal and pelvic ultrasound revealed a large mass (14 × 8.6 cm) that was continuous with the uterus, containing non-pure, echogenic liquid suggestive of vaginal distension with hemorrhagic content, as well as slight left-sided hydronephrosis (renal pelvis measuring 10 mm) caused by compression. No genitourinary or anorectal malformations were observed (Figure 1). Laboratory testing did not show any increased inflammatory parameters or abnormal kidney function. Urinalysis was normal.

After confirming the diagnostic hypothesis, the patient was referred to pediatric surgery for a hymen incision, which was performed under sedation and cardiorespiratory monitoring. The procedure consisted of a cruciform incision on the hymen and excision of redundant tissue with drainage of a large amount of hemorrhagic content.

Within a few hours, there was a complete resolution of symptoms, with granted hymen permeability and no complications. The adolescent was discharged from the hospital within 48 hours. At the pediatric surgery follow-up, five months later, the adolescent remained asymptomatic with regular menstrual cycles. Revision ultrasound showed complete resolution of the hydronephrosis.

## Discussion

Hematocolpos is an infrequent condition characterized by the accumulation of menstrual blood in the vagina, instead of its normal elimination. It can be caused by several uterovaginal deformities including imperforate hymen, transverse vaginal septum, vaginal atresia, hemivaginal atresia, cloacal malformations and acquired vaginal stenosis [5,6]. Imperforate hymen accounts for 90% of the cases of hematocolpos and is the most common anomaly of the female genital tract. Although limited epidemiological data are available, the estimated prevalence of imperforate hymen is 1:2000 girls [5,7,8].

The development of the female genital tract is a complex process that begins at three weeks of embryogenesis and involves multiple successive steps. The embryological origin of the lower third of the vagina is the urogenital sinus, suggesting that the hymen is of non-Müllerian origin. The hymenal membrane consists of fibrous connective tissue surrounded by stratified squamous epithelium which separates the vaginal lumen from the urogenital sinus. Rupture of the hymen usually occurs before birth due to the degeneration of the central epithelial cells of the hymen, leaving a thin fold of mucous membrane remaining around the vaginal introitus. When the canalization of the vaginal plate is unsuccessful and the degeneration of the hymenal epithelial cells fails, it results in an imperforate hymen [5,6].

The diagnosis of imperforate hymen can be established in the neonatal period, with the presence of mucocolpos, an accumulation of vaginal secretions due to stimulation by maternal estrogen which causes a bulging introitus noted at birth. When this does not occur, mucus is absorbed, and the child remains asymptomatic until adolescence. Symptoms typically manifest during puberty and are often nonspecific, including cyclic lower abdominal or pelvic pain in an adolescent with primary amenorrhea.

Hematocolpos refers to the accumulation of menstrual blood in the vagina, and hematometra to blood in the uterus. Significant distension of the vagina may also cause back pain, pain with defecation or difficulties with voiding. The physical examination should include perineal inspection, which may reveal a bulging and bluish hymen.

The diagnosis of this condition can be challenging and delayed due to its variable clinical manifestations. As described in this case, the hematocolpos can cause mechanical obstruction of the urinary tract, resulting in urinary retention and hydronephrosis. In some cases, it can also lead to acute renal injury. The differential diagnosis of imperforate hymen includes other obstructive anomalies of the vagina that can also cause a bulging mass introitus, such as agenesis of the lower vagina and a low transverse vaginal septum. Performing an ultrasound is essential to establish the diagnosis and proceed with adequate surgical treatment. If the genital examination presents a bulging mass in the vaginal introitus suggestive of an imperforate hymen and the ultrasound confirms the diagnosis of hematocolpos, no further imaging is required. However, if the diagnosis is unclear, an MRI is recommended to exclude other urogenital anomalies [9].

Hymen incision is mandatory, with full blood drainage, excision of redundant hymen edges (as it was performed in our case), or hymenectomy [9]. Delays in diagnosis and treatment can lead to serious complications such as tubal infection and adhesion, pelvic endometriosis, infertility, and renal failure secondary to hydronephrosis [6]. The prognosis is favorable with an early diagnosis, allowing timely successful drainage of an imperforate hymen.

## Conclusions

This case shows a less frequent presentation of hematocolpos, with initial symptoms suggestive of lower urinary tract infection and constipation. Definite diagnosis was also a challenge due to the absence of a typical cyclic abdominal pain. It is crucial to consider hematocolpos in the differential diagnosis of acute urinary retention in a female adolescent without prior menarche and to include the evaluation of the pubertal development (Tanner staging) and genital inspection in the physical examination. Timely diagnosis and treatment can prevent complications and preserve fertility in these patients.
